# Medicinal Mushrooms, Probiotics and Combination of Natural Compounds in the Management of HPV: A Comparative Look at Viral Clearance and Lesion Resolution

**DOI:** 10.3390/v17070942

**Published:** 2025-07-02

**Authors:** Giuseppina Porcaro, Marco Calcagno, Andrea Tinelli

**Affiliations:** 1 Department of Gynecology and Obstetrics, Women’s Health Centre, 05100 Terni, Italy; giusy.porcaro@gmail.com; 2 Department of Woman Health and Reproductive Medicine, Santo Spirito Hospital, 00193 Rome, Italy; 3 Department of Obstetrics and Gynecology, CERICSAL (CEntro di RIcerca Clinico SALentino), Veris delli Ponti Hospital, 73020 Scorrano, Italy

**Keywords:** HPV, natural molecules, dietary supplements, epigallocatechin gallate, folic acid, vitamin B12, hyaluronic acid, medicinal mushroom, probiotics

## Abstract

Despite the fact that human papillomavirus (HPV) infections are common, there are currently no proven treatment approaches for persistent infections. A promising strategy to promote HPV clearance and the regression of caused lesions is dietary supplementation with natural compounds. This review evaluates available supplement formulations proposed in HPV care, focusing on combinations of epigallocatechin gallate (EGCG), folic acid (FA), vitamin B12 (B12), and hyaluronic acid (HA), as well as medicinal mushrooms and probiotics. The combination of EGCG, FA, B12, and HA is supported by the most consistent evidence, which shows a high rate of HPV clearance and lesion resolution across several clinical investigations. Medicinal mushrooms and probiotics have also shown some evidence of beneficial effects, although the diverse designs of the reported clinical studies may limit the observed findings. Overall, natural molecule-based supplements showed promising safety and efficacy profiles in the management of HPV persistent infection, supporting their clinical use. Of course, further investigations through well-designed, large-scale, randomized controlled trials will be necessary to provide strong support.

## 1. Introduction

The human papillomavirus (HPV) is a double-stranded DNA virus belonging to *Papillomaviridae* family, responsible for the most common sexually transmitted infection worldwide [1] and the primary cause of cervical cancer [2]. Actually, beyond the cervical region, HPV infection may also involve anal and oral anatomical sites. In particular, emerging evidence indicated a rising prevalence (7.4%) of oral HPV infection, particularly in men across different geographic locations (i.e., the US, France, Germany, Spain, and the UK) as reported in a recent cross-sectional study involving over 7600 participants [3]. Recent publications identified more than 150 strains of HPV, which can be divided into low-risk (LR) or high-risk (HR) according to their own oncogenic potential [4,5].

In particular, LR-HPVs principally cause genital warts and rarely correlate with cancer, while infections due to HR-HPVs can cause the development of cancer in the uterine cervix, vulva, vagina, penis, and head–neck regions. Several risk factors have been considered when addressing the pathogenesis of HPV. The majority are associated with sexual intercourse (i.e., early sexual activity, having multiple sex partners, inconsistent protection) and/or comorbidities (i.e., HIV infection, compromised immune system) as well as education, lifestyle (i.e., unhealthy lifestyle, such as smoking or excessive alcohol consumption), and socioeconomic status [6,7,8,9,10].

The immune system can play a crucial role in counteracting the infection within 1 to 2 years [11,12], with over 90% of them resolved within 7 years [13]. However, the clearance rate of HPV may significantly vary according to age, sex and ethnicity as reported by several longitudinal studies [14,15,16]. As a matter of fact, in a study published in *Lancet* involving over 1000 males, 66% exhibited HPV clearance at 12 months, increasing to 90% by 24 months [17]. However, when infection persists, HPV DNA may integrate into the host genome and predispose it to tumoral development. From a molecular point of view, the HPV genome encodes two key oncogenic proteins, E6 and E7, which are able to degrade the tumor suppressor protein p53 and the retinoblastoma protein (pRb), respectively [2,18]. As a consequence, this dual inactivation disrupts cell cycle checkpoints, promotes uncontrolled proliferation, and induces genomic instability, driving toward tumoral progression, a process sustained by the persistent upregulation of HPV E6/E7 oncogenes [19,20]. The management of persistent infection represents a great challenge in the management of HPV. In the past few years, several definitions of persistent HPV infection have been proposed, primarily based on genotype-specific infection duration and influenced by factors such as testing frequency and interval length. While some studies define HPV persistence as the detection of the same HPV genotype at two consecutive time points (i.e., in tests conducted 6 months apart), others adopt stricter criteria, requiring consistent detection across three or more intervals [21,22,23,24].

HPV infection correlates with the occurrence of cytological lesions of varying severity that undergo different medical approaches. Most cervical low-grade lesions (LSIL/CIN1) can spontaneously regress (90%) without the need for invasive treatments [25], so patients with such cytological lesions usually undergo a “wait and see” approach. However, when low-grade lesions persist over years of HPV infection, they may progress toward more serious lesions that need medical intervention. Indeed, cervical high-grade lesions (HSIL/CIN2-CIN3) are more likely to persist (32%) and progress (12%) into cancer if untreated [26,27]. This risk is particularly elevated among immunocompromised individuals or those with specific genetic susceptibilities [28]. Ablative or excisional procedures are the standard treatment in the management of patients with HPV and HSIL. Nevertheless, a considerable percentage of women (from 10% to 53%) undergoing these surgical excisions (LEEP) still experience persistent disease [29,30].

Concerning the management of the infection, vaccination remains the most effective method for preventing HPV infection, especially against HR genotypes (i.e., HPV 16 and 18), while screening programs may help to detect viral positivity and lesions early, thus preventing the progression of precancerous lesions into carcinoma [31,32,33].

There is still no proven cure for HPV, especially for people with persistent HR-HPV infections, despite advancements in scientific study and the introduction of screening programs. Scientific research has explored a number of compounds in an effort to develop new treatment approaches to combat HPV infection and its persistence, given the intricacy of the pathogenesis. In recent years, scientists have become more interested in the natural bioactive compounds found in dietary supplements. These substances have antioxidant, immunomodulatory, and antiviral properties that may help alleviate HPV-related symptoms and encourage virus removal with varying degrees of effectiveness.

**Figure 1:**
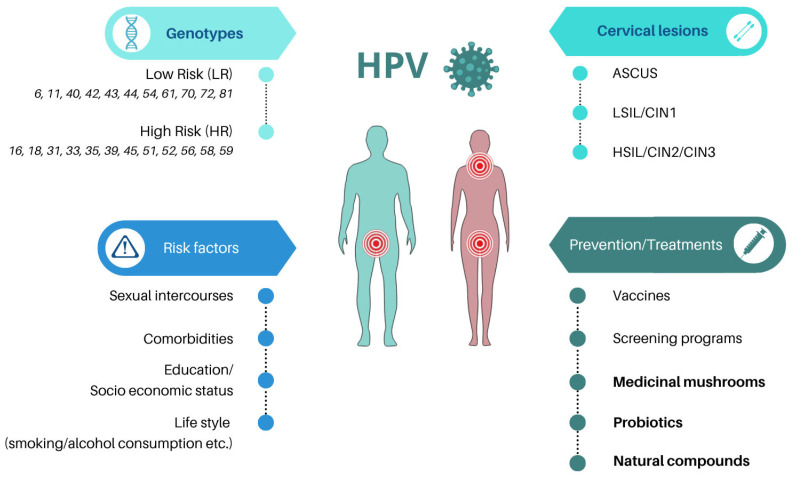
Overview of HPV infection characteristics. This figure provides a visual summary of key aspects of HPV infection, including anatomical sites commonly affected (oral, anal/rectal, cervical/vaginal, and penile), classification of HPV strains into low-risk (LR) and high-risk (HR) types, associated premalignant and malignant cervical lesions, risk factors for infection and persistence, as well as preventive and treatment strategies.

In this review, we have collected evidence about available supplement formulations containing natural molecules currently proposed in the management of HPV infection, with a focus on those strategies that are incorporated into products intended for patient care (Figure 1).

## 2. Materials and Methods

The presented narrative review uses a non-systematic search to evaluate the efficiency of available supplement formulations and their potential as therapeutical options for HPV. Relevant studies were selected using the following databases or Gen AI web tools: PubMed (MEDLINE), Scopus, Google Scholar, Gemini (Gen AI), and Perplexity (Gen AI). The following keywords—“HPV”, “HPV infection”, “HPV clearance”, “HPV regression”, “HPV lesions”, “HPV persistence”—were combined with (AND/OR) “supplements”, “dietary supplements”, “natural molecules”, “combination of natural compounds”, “epigallocatechin gallate”, “folic acid”, “vitamin B12”, and “hyaluronic acid”, “fungi”, “medicinal mushrooms”, “mushrooms”, “microbiota”, “lactobacillus”, “antioxidant”, “plant”, “plant extract”, “multi-target agents”, and “synergistic effects”. Papers were further filtered by English language, while no restrictions were applied to the year of publication. After the careful screening of content based on titles and/or abstracts, the articles were organized based on supplement category type (i.e., probiotics, medicinal mushrooms, and natural compounds) and reported outcomes (i.e., HPV clearance and lesion resolution after treatment). Studies involving populations with comorbidities such as HIV or other immune-compromising conditions as primary selection criteria were explicitly excluded to ensure a more homogeneous evaluation of the effects of the supplements on HPV individuals. Studies lacking relevance to the research question were excluded.

## 3. Dietary-Based and Local Approaches in the Management of HPV Infection

**Figure 2:**
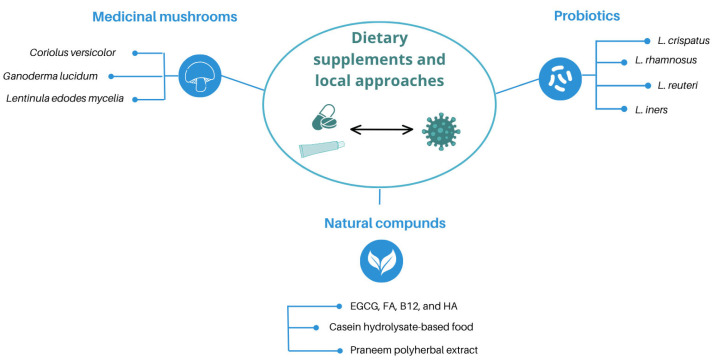
Overview of dietary supplementation-based and local vaginal strategies for counteracting HPV infection. Medicinal mushrooms, probiotics, and combinations of natural compounds reflect the most commonly used approaches in HPV management.

Dietary supplementation based on micronutrients and natural molecules and local vaginal approaches have received increasing attention in the management of HPV infection. Medicinal mushrooms, probiotics, and combinations of natural compounds represent strategies with varying efficacy in promoting HPV clearance and the resolution of induced lesions (Figure 2; Table 1).

### 3.1. Medicinal Mushrooms

Medicinal mushrooms have largely been utilized for thousands of years in traditional medicine due to their therapeutic or prevention properties. In the past 10 years, the use of medicinal mushrooms, such as *Coriolus versicolor*, *Ganoderma lucidum*, *Shiitake*, *Schisandra chinensis*, and *Pueraria lobata*, has gained interest for their role in promoting HPV clearance.

However, among the various mushroom-based formulations described, *Coriolus* and *Shiitake* have attracted increasing interest due to their effect in counteracting the infection of HPV.

*Coriolus versicolor*, also known as *Trametes versicolor* or turkey tail mushroom, is a medicinal fungus recognized for its immune-modulating properties and potential therapeutic effects against various antimicrobial and viral infections [34]. A preliminary randomized, controlled clinical trial conducted by Donatini and colleagues demonstrated that the 2-month oral administration of 200 mg/day of *Coriolus versicolor* and *Ganoderma lucidum* resulted in an 88% clearance rate of oral HPV infection, among the initial 41 positive patients for HPV16 and HPV18, which was statistically significantly higher than the 5% clearance rate observed in the control group [35]. However, no clinical studies have been reported for *Ganoderma lucidum* as standalone treatment for HPV cervical infection contrary to *Coriolus versicolor*.

Five clinical studies have evaluated a 21-day regimen (repeated monthly for 3 months) of *Coriolus versicolor*-based vaginal gel in HR-HPV positive women, including three from the PALOMA/PAPILOBS trial series. In particular, this gel contains *Coriolus versicolor* extract rich in polysaccharopeptides (PSP), bioactive compounds known for their potent immunomodulatory properties. PSP has been shown to stimulate the production of pro-inflammatory cytokines, activate immune cells such as macrophages and natural killer (NK) cells, and enhance antigen presentation, contributing to its potential role in promoting antitumor immunity [34].

In a longitudinal retrospective observational study carried out by Criscuolo and colleagues, such formulation was associated with a statistically significant HR-HPV clearance in 67% of cases and lesion resolution in approximately 77% of cases after 6 months, compared to respective control groups [36].

The first study from the PALOMA trial was a multicenter, open-label, randomized, parallel-group, controlled clinical trial that enrolled 90 HPV-positive patients presenting cervical LSILs. After 3 months, lesion regression was observed in 78% of treated patients compared to 54.8% of untreated controls, increasing to 85% versus 64.5%, respectively, after 6 months, with both differences being statistically significant. However, the overall HPV clearance was not statistically significant when compared to controls after 6 months of treatment [37].

In a subsequent sub-analysis of the same PALOMA trial conducted in women older than 40 years old, *Coriolus versicolor*-based treatment contributed to restoring low-grade cervical–vaginal lesions in 24 women out of 26, while 6 women out of 12 spontaneously resolved in a 6-month period. Consistent with earlier findings, no significant differences were observed in the obtained viral clearance between treatment (61.5%) and control (50%) groups [38]. Finally, an enlarged multicentric real-world study (PAPILOBS trial), conducted without a control group and enrolling 192 participants, reported that 67% of patients with cervical lesions (ASCUS/LSIL) showed restored altered cytology after 6 months of treatment with *Coriolus versicolor*-based vaginal gel, increasing to approximately 77% within 12 months of treatment. Regarding HPV clearance, about 59% of women tested negative after 6 months of treatment, which rose to 72% within 12 months of treatment [39].

Overall, *Coriolus versicolor*-based vaginal gel demonstrates high patient adherence, with rates ranging from 93 to 98% in long-term use [37,39], although it was not explicitly reported in others [36,38]. Concerning tolerability, mild adverse events (i.e., vulvovaginal itching/stinging) were reported in only 3 patients by Cortès Bordoy and colleagues [39]. However, a higher incidence was observed by Serrano and colleagues [37], with 7 out of 17 reported adverse events considered possibly or probably related to the treatment, most of which were mild or moderate in severity, and 3 cases requiring temporary or permanent discontinuation. Even though the impact on quality of life was not assessed in all reported studies, this treatment seems to be positively associated with improvement in quality of life, as demonstrated by the reduction of perceived stress in two studies [37,39].

*Lentinula edodes mycelia*, derived from *Shiitake* mushroom, is a medicinal fungus known for its immune-enhancing, antitumor, and antiviral properties [40]. Traditionally utilized in Asian medicine, it has gained attention in modern research for its bioactive compounds. Extracts from *Lentinula edodes mycelia* have demonstrated antiviral activity, including the direct inhibition of the influenza virus replication during early stages of infection [41]. Notably, the polysaccharides present in *Lentinula edodes* act as immunomodulators and biological response modifiers by activating macrophages, NK cells, dendritic cells, and T lymphocytes, as well as by promoting the production of cytokines such as Interferon-gamma (IFN-γ), Interleukin 12 (IL-12), and Tumor necrosis factor alpha (TNF-α). These effects contribute to the enhancement of both innate and adaptive immune responses [42].

Two studies conducted by Smith and colleagues evaluated the effect of the oral administered standardized extract of *Lentinula edodes mycelia* on HPV clearance. The first was a pilot uncontrolled study involving women with persistent HR-HPV infection [43], while the second a phase II, randomized, double-blind, placebo-controlled trial [44]. In both studies, which differ in dosage regimens (1 and 3 g/day) and number of enrolled patients (6 and 41), HPV clearance rate ranging from 44 to 67% was observed over treatment periods of 3 to 8 months [43,44]. Differently from the first study, which lacked a control group, the second study observed a 10.5% spontaneous clearance rate in the placebo group after 12 months [44]. Although quality of life was not reported in either study [43,44], patient adherence was monitored through pill diary reviews every 3 months during clinic visits [44]. In terms of tolerability, no significant adverse effect was reported, aside from single cases of nausea and bloating.

Considering the use of *Coriolus versicolor*-based vaginal gel in HPV management, none of the studies reported a stratification of the outcomes according to potential confounding variables, such as BMI, sexual activity, smoking, and use of hormonal pills.

Despite the positive results regarding the use of the above-mentioned medicinal mushrooms in the management of HPV infection, several methodological and design-related constraints may resize the effective strength of these findings. In particular, most of the studies present a small number of enrolled patients or the lack of an appropriate control group. Moreover, many of the reviewed studies do not provide clinical data at the 3-month timepoint, which represents a critical window for assessing early virological and cytological changes.

### 3.2. Probiotics

The use of probiotics in the management of HPV has gained increasing interest in recent years, considering their role in enhancing immune function, maintaining a healthy vaginal microbiome, and reducing the risk of secondary infections. At the physiological level, cervicovaginal microbiota is mostly represented by *Lactobacillus* species, which play a key role in maintaining a protective microenvironment against infection [45]. Indeed, the use of *Lactobacilli* offers a broad spectrum of protection via different mechanisms: (i) by modulating the immune system; (ii) by mediating microbial competition; and (iii) by producing metabolites (e.g., lactic acid, bacteriocins, and biosurfactants). In this context, several studies explored the combination of probiotic-based approaches either alone or in combination with conventional therapies for HPV management. For instance, IFN-based therapy, commonly used in antiviral and immunomodulatory treatments [46], has been investigated in combination with vaginal *Lactobacilli*-enriched probiotics, resulting in the recovery of HPV infection in 29% of cases [47].

Among *Lactobacillus* species, *L. crispatus* exerts a crucial role in protecting the female reproductive tract. A vaginal microenvironment enriched in *L. crispatus* can inhibit the growth of various vaginal pathogens, including HPV, by preventing its integration into the basal keratinocytes. Along with this, some other *Lactobacillus* species like *L. gasseri* and *L. casei* may positively affect HPV clearance [48,49]. On the other hand, *L. iners* has been commonly associated with a lower HPV clearance rate due to its ability to survive under metabolic-stress conditions and its limited effectiveness in preventing pathogen colonization [50]. Given the association between a *Lactobacillus*-rich vaginal microbiota and a reduced risk of HR-HPV infection and cervical disease, probiotics are being explored as a strategy to restore microbial balance in HPV-positive women [51,52]. Several evidence reported that the modulation and/or the restoration of microbiota dysbiosis can contribute to significant health benefits, including enhanced HPV clearance and improvement in cytological abnormalities. A study by Di Pierro and colleagues enrolled 35 HPV-positive women, most of whom were characterized by a vaginal microenvironment depleted in *Lactobacillus* species. The authors observed 70% viral clearance after 3 months of oral treatment with *L. crispatus*, along with an improvement in the vaginal microenvironment [53]. Considering the limitations of the study due to its open, non-controlled design, the authors demonstrated that the oral assumption of *L. crispatus* M247 is able to restore vaginal microbiota and improve HPV clearance [53]. In contrast, a pilot randomized, untreated controlled retrospective trial by Dellino and colleagues found no significant differences in HPV clearance among 80 women with low-grade cervical lesions who have received *L. crispatus* supplementation for 12 months, compared to a control group. While no changes were observed after 6 months, the improvement or regression of cytological low-grade lesions was significantly greater in the treated group (60.5% of cases) compared to controls (41.3% of cases) at the end of the treatment [54].

Taken together, these findings suggest that while *L. crispatus* has a potential role as a supportive intervention for improving HPV clearance, further research is needed to clarify its efficacy and define its role in clinical practice.

Along with *L. crispatus*, a combination of both *L. gasseri* and *L. casei* probiotics contributes to modulate the vaginal microbiome and enhances the natural clearance of HPV. A prospective, controlled pilot study by Verhoven and colleagues demonstrated that 6-month treatment with a probiotic drink containing *L. casei* strain Shirota was associated with statistically significant improvement in cytological regression among women with LSIL, compared to the untreated control group (60% vs. 31%). Additionally, an increased likelihood of HPV clearance was observed in the probiotic group compared to the untreated group (29% vs. 19%), although the difference was not statistically relevant [55].

Other interesting results have been observed for *L. rhamnosus* as either a standalone or an adjunctive treatment. Indeed, *L. rhamnosus* plays a beneficial role in gut health by strongly adhering to intestinal mucus through its SpaCBA pili proteins and lipoteichoic acid, modulating the immune system, promoting epithelial integrity, and producing antimicrobial substances that help inhibit pathogenic bacteria [56]. A randomized controlled clinical study involving 117 HPV-positive patients with bacterial vaginosis demonstrated that those receiving standard treatment for bacterial vaginosis combined with the vaginal administration of *L. rhamnosus BMX 54* exhibited a statistically significant increase in HPV clearance after 6 months compared to those treated for 3 months [57].

As for *L. rhamnosus*, *L. reuteri* is known to exert a beneficial effect through multiple mechanisms, including the production of antimicrobial substances such as reuterin, organic acids, and ethanol, which help inhibit the growth of pathogenic bacteria. It also contributes to enhancing tight junction protein expression, reducing bacterial translocation, and modulating gut inflammation [58].

Furthermore, a randomized clinical trial conducted by Ou and colleagues evaluated the effect of orally administered *L. rhamnosus GR-1* and *L. reuteri RC-14* strains in 62 women with HR-HPV infection. The authors revealed that such supplementation did not influence HR-HPV clearance, with a clearance rate of 58% in the treated group compared to 54% in the control group [59].

A preliminary prospective observational study on a small cohort of 15 HPV positive women with LSILs used a combined approach based on both *L. crispatus* M247 and *Shiitake* mushroom supplementation. The authors observed a statistically significant clearance rate of approximately 73% in the treated group over a 6-month period compared to the untreated control group. Moreover, while no complete resolution of LSILs to normal cytology was observed, a significant regression to chronic cervicitis occurred in 13% of cases in the intervention group after 6 months compared to the control [60].

Among the selected probiotic-based interventions, good tolerability with no significant adverse events, aside from mild nausea observed in 2 patients, was reported only in a single study [54]. Concerning the patients’ adherence, the only study reporting excellent compliance (98%), with almost all participants attending the follow-up visits, was Dellino et al. [54]. Palma et al. [57] reported that all patients followed a strict follow-up, although general adherence to the treatment regimen was not specifically reported. In contrast, adherence was not evaluated in the studies by Ou et al. [59], Verhoeven et al. [55], or Salimbeni et al. [60].

Overall, none of the described studies specifically stratify the observed outcomes according to confounding variables, such as BMI, smoking status, alcohol consumption, sexual activity, and use of hormonal contraceptives. However, only one study acknowledged, as a limitation, the lack of assessment of variables such as smoking, sexual activity, use of oral contraceptives, and vaginal pH as potential modifiers of the observed outcomes [59].

The possibility of modulating vaginal microbiota to counteract HPV infection and its persistence seems interesting, considering the crucial role of vaginal dysbiosis in exposure to the risk of developing HPV persistence and tumoral progression. However, most of the reported studies draw controversial findings and lack important considerations. In line with this, a recent systematic review published in *Lancet Microbe*, which analyzed data from 105 reports, highlighted that even though some studies suggest a potential benefit of probiotics, particularly *L. crispatus*, *L. rhamnosus*, and *L. reuteri*, in restoring vaginal microbiota and supporting HPV clearance, the overall quality of evidence remains low due to small sample sizes and heterogeneous study designs. Furthermore, the review underscored the lack of evidence supporting the hypothesis that the manipulation of the vaginal microbiota can influence the microbial composition of other compartments within the female genital tract [61]. In addition, clinical studies reported limited information regarding the resolution of cervical lesion post-treatments, which makes it difficult to assess the clinical relevance of these interventions in HPV management.

### 3.3. Natural Compounds

In recent years, several natural molecules have emerged as promising agents in enhancing viral clearance and resolving HPV-induced lesions. Some of them include Praneem polyherbal and casein hydrolysate formulations. The only available report for Praneem comes from a non-randomized, open-label, placebo-controlled pilot study published in 2009. This study demonstrated HPV clearance in 60–80% of treated patients as well as the amelioration of pre-neoplastic lesions and cytological abnormalities in a small group of patients following 1–2 months of intra-vaginal application, compared to a spontaneous clearance rate of 10% in the control arm. Notably, the authors reported no adverse effects after the application of vaginal gel during the study period [62].

Concerning the casein hydrolysate formulation, a recent prospective, non-interventional observational study reported a higher HPV clearance rate (ranging from 40 to 68%) after 6 months of treatment, even though it did not achieve a statistically significant difference when compared to the untreated control group. Regarding tolerability, almost all patients (95%) reported no adverse effects, with only one experiencing mild abdominal swelling. Additionally, 50% of participants reported general improvements, including a lower incidence of cold, increased vitality, and fewer recurrences of cold sores [63].

In addition, two herbal extracts derived from Asian traditional medicine, a Chinese multiherb tincture (Gaoweikang) and Yokuinin, have also been mentioned in the literature for their potential role in HPV management [64,65]. However, their clinical application remains limited, warranting further research to assess their efficacy, safety, and potential for standardization.

Besides this, among the studied natural molecules for their antiviral effect, the combination of epigallocatechin gallate (EGCG), folic acid (FA), vitamin B12 (B12), and hyaluronic acid (HA) has been extensively investigated in the management of HPV in recent years. Among these, EGCG, one of the most well-known bioactive molecules contained in green tea, has immunostimulatory, antioxidant, antiproliferative, and anticancer properties [66], with the ability to suppress E6/E7 oncogene expression, thus promoting apoptosis. Interestingly, Frega and colleagues first demonstrated the additive effect of such molecules in stimulating apoptosis in a model of HPV-infected Hela cells [67]. Their study showed that the combination of EGCG, FA, B12, and HA significantly increased apoptosis compared to each compound alone, an effect associated with the upregulation of p53 expression and the downmodulation of HPV E6/E7 viral oncogenes. Subsequently, five clinical studies investigated the antiviral effects of the combination of EGCG, FA, B12, and HA in relation to HPV infection. Two publications address clinical cases about the therapeutic effect of EGCG, FA, B12 and HA in HPV persistent infections. The first case report on a young, fertile woman with a 9-year history of HPV persistence, who had undergone multiple surgical interventions, demonstrated that after only two months of treatment based on two tablets per day of such combined molecules, preexisting HSIL and LSIL lesions significantly improved. In addition, a 6-month period of follow up confirmed the negativity to HPV infection [68]. A subsequent publication reported the improvement of HPV persistent infection in five clinical case reports, considering also a case of anal HPV infection. The oral treatment with one or two tablets per day for 6 or 3 months, respectively, according to the individual clinical history of the patient, revealed HPV clearance in all 5 patients, also resolving pre-existing cervical lesions [69]. Even though these results regard only a small number of patients, other clinical studies reported the same evidence on larger populations.

A pilot, open-label, controlled clinical trial by Aragona and colleagues evaluated the effect of treatment on 40 women (20 each in treatment and untreated control groups) with HPV persistence of two or more years and LSILs, observing a statistically significant viral clearance rate and lesion regression in 85% of treated patients, compared to 25% in the control group, after 3 months. Interestingly, among the remaining 15% who did not respond to the treatment, only a single person reported LSIL, while the other two patients reported ASCUS [70]. Moreover, another open-label, controlled clinical trial conducted by Tinelli and colleagues on 163 women demonstrated that approximately 81% of patients in the treated group achieved HPV clearance after 3 months, compared to around 60% in the untreated control group evaluated at the same timepoint, a difference that was statistically significant. The treatment also significantly improved cervical low-grade lesions in about 41% of treated patients compared to 9% of cases in the control group. In addition, after 6 months, a significant improvement in cytological results was observed in approximately 84% of treated patients, compared to about 37% in the control group [71].

Finally, a recent single-arm, open-label clinical study involving a larger number of patients (*n* = 106) confirmed that a 6-month treatment significantly both cleared the viral infection and resolved baseline lesions in about 86% and 92% of patients, respectively. By analyzing the obtained results across patients’ age, the authors observed a complete clearance in younger women (26–35 years old) or a nearly total clearance (91%) in older women (>46 years old). A lower but still statistically significant clearance rate was observed in the 36–45 age group (69.4%). Moreover, preliminary data at the 3-month timepoint indicated the promising early efficacy of the oral supplementation based on EGCG, FA, B12, and HA, with a statistically significant viral clearance rate observed in approximately 67% of patients and lesion resolution in 80% [72].

The evaluation of patients’ adherence and tolerability was also assessed in some of the reported studies regarding the combination of EGCG, FA, B12, and HA. Concerning the patients’ adherence, three studies showed complete adherence [68,69,72], although not explicitly reported. Regarding tolerability, Porcaro et al. [72] reported no adverse effects in any of the tested patients, while Aragona et al. [70] documented a minor gastrointestinal disturbance that resolved without discontinuing the treatment in the single patient case.

Overall, none of the reported studies on the combination of EGCG, FA, B12, and HA in HPV management stratified their findings according to potential confounding variables such as age, BMI, smoking status, number of sexual partners, or contraceptive pill use. However, in the studies conducted by Tinelli et al. [71] and Aragona et al. [70], patients were evenly allocated between treatment and control groups with respect to key variables including sexual activity (i.e., number of partners), smoking status, and BMI, in order to minimize potential confounding effects and ensure comparability between groups.

Among the reported treatments with natural compounds, the combination of EGCG, FA, B12, and HA natural extract revealed high rates of HPV clearance and lesion resolution, thus suggesting potential applicability as a promising therapeutic approach promoting HPV clearance and lesion regression.

**Table 1:** Comparison of clinical studies using dietary supplementation-based and local approaches on HPV clearance and lesion resolution.

Dietary Supplementation Treatment	Outcomes		Dosage	Reported Limitations	Duration of Treatment	Sample Size	Study Design	HPV Genotypes
	HPV Clearance (%)	Lesion Resolution (%)						
**Medicinal mushrooms**								
*Coriolus versicolor + Ganoderma lucidum* [35]	88% (*n* = 36/41)	N/A	Two capsules of 200 mg/day	Limited sample size	2 months	61 (41 treated; 20 controls)	Randomized, controlled clinical trial	HR (HPV 16, 18) genotypes
*Coriolus versicolor* [36]	67% (*n* = 65/97)	77.3% (*n* = 75/97)	Vaginal gel application (1 cannula/day for 21 days; repeated for 3 months)	Bias derived from non-experimental study design	6 months	183 (97 treated; 86 controls)	Longitudinal retrospective observational, controlled trial	HR genotypes (no additional specification)
*Coriolus versicolor* [37]	N/A	78% (*n* = 46/59)	Vaginal gel application (1 cannula/day for 21 days; repeated for 3 months)	Lack of biopsies, unblinded assessments, and missing data on cofactors like smoking	3 months	91 (59 treated; 32 controls)	Open-label, randomized, controlled trial	HR (16, 18, 31, 33, 35, 39, 45, 51, 52, 56, 58, 59, and 68) genotypes
	59.6% (*n* = 31/52)	84.9% (*n* = 45/53)			6 months			
*Coriolus versicolor* [38]	61.5% (*n* = 16/26)	92.3% (*n* = 24/26)	Vaginal gel application (1 cannula/day for 21 days; repeated for 3 months)	Limited sample size (especially in HR-HPV 16-18-31 strains)	6 months	38 (26 treated; 12 controls)	Open-label, randomized, controlled trial	LR (not specified) and HR (6, 18, 31, 33, 35, 39, 45, 51, 52, 56, 58, 59 and 68) genotypes
*Coriolus versicolor* [39]	58.7% (*n* = 78/189)	67% (*n* = 128/191)	Vaginal gel application (1 cannula/day for 21 days; repeated for 3 months)	Absence of a control arm, high number of drop-out patients, lack of info on HPV-related cofactors, lack of analysis of regression and clearance for specific HPV genotypes	6 months	192 (all treated)	Multicentric real-world study	LR (not specified) and HR (16, 18, 31, 33, 35, 39, 45, 51, 52, 56, 58, 59, and 68) genotypes
	71.6% (*n* = 54/190)	77.1% (*n* = 148/192)			12 months			
*Lentinula edodes mycelia (Shiitake)*	66.7% (*n* = 4/6)	N/A	3 g/day	N/A	3–6 months	10 reported (6 analyzed)	Pilot uncontrolled study	HR genotypes (not specified)
Trial 1 and 2 [43]	44% (*n* = 4/9)	N/A	1 g/day	N/A	>8 months	10 reported (9 analyzed)		
*Lentinula edodes mycelia* (*Shiitake*) [44]	63.6% (*n* = 14/22)	N/A	3 g/day	Limited sample size, single-arm analysis	6 months	34 (22 treated; 12 controls)	Randomized, double-blind, placebo-controlled trial	HR genotypes (not specified)
**Probiotics**								
*L. crispatus* [54]	N/A	20%	*L*. *crispatus* M247 (2 × 10^10^ CFU) for 12 months	No statistical differences between groups for HPV clearance; lack of specific microbiota community analysis	6 months	160 (80 treated; 80 conrols)	Randomized, untreated controlled retrospective trial	HR (HPV 16, 18, 66, 68, 58, 45, 53, 51, 52, 35); LR (HPV 6, 11, 40)
	15.3%	61.5%			12 months follow-up			
*L*. *crispatus* M247 [53]	70% (*n* = 24/35)	N/A	N/A	Open, non-controlled study	3 months	35 (all treated)	Open-label, non-controlled clinical study	HPV+ (Not specified)
*L. rhamnosus BMX 54* [57]	11.6% (*n* = 7/60)	37.5% (*n* = 15/40)	Vaginal tablet containing 10^4^ CFU *L. rhamnosus* (once every 3 days for initial 20 days + once a week until 3/6 months)	Lack of controls	≤3 months	117 (60 short treatment; 57 long treatment)	Randomized controlled clinical study	HR (16, 18, 31, 33, 35, 39, 45, 51, 52, 56, 58, 59 and 68)
	31.2% (*n* = 18/58)	79.4% (*n* = 31/39)			Follow-up median 14 months (range 9–30 months)			
*L. rhamnosus GR-1* and *L. reuteri RC-14* [59]	58.1% (*n* = 36/62)		One caps/day (≥10^9^ CFU/g of each strain)	Low statistical power (55.8%); loss to follow-up; no significant impact on HPV clearance rates	3–12 months (total clearance)	121 (62 treated; 59 controls)	Randomized clinical trial	HR genotypes (Not specified)
	33.1%	ns			3 months			
	43.8%	41.9% (*n* = 26/62)			6 months			
	47.9%	N/A			9 months			
	56.2%	N/A			12 months			
*L. casei Shirota* [55]	25%	N/A	Daily probiotic drink during the study period (6 months)	Heterogeneity among participants (e.g., age, duration of infection. Lack of randomization; absence of statistical power	3 months	51 (24 treated; 27 controls)	Prospective, controlled (untreated) pilot study	HR genotypes (16, 18, 26, 31, 33, 35, 39, 45, 51, 52, 53, 58, 59, 66, 68, 73, and 82)
	29.2%	60% (*n* = 12/20)			6 months			
**Probiotics + Mushroom**								
*L*. *crispatus* M247 + *Lentinula edodes mycelia (Shiitake)* [60]	73.3% (*n* = 11/15)	N/A (regression to chronic cervicitis in 13% of cases; n = 2/15)	6 caps (500 mg each)/day + *L*. *crispatus* M247 (2 × 10^10^ CFU)	Sample size; lack of randomized groups	6 months	34 (15 treated; 19 controls)	Prospective observational study	LR (HPV-6, -11, -40, -42, -43, -44, -54, -61, -70, -72, -81); HR (HPV-16, -18, -26, -31, -33, -35, -39, -45, -51, -52, -53, -56, -58, -59, -66, -68, -73, -82) genotypes
**Natural extracts**								
Praneem (Azadirachta indica + Sapindus muckerossi + Mentha citrate + Quinine Hydrochloride)	60% (*n* = 6/10) 80% (*n* = 8/10)	N/A N/A	500 mg/day 500 mg/day	N/A	1 month 2 months	20 (10 treated; 10 controls)	Non-randomized, open-label, placebo-controlled pilot study	HR (HPV 16) genotype
[62]								
Casein hydrolysate-based food [63]	50.7% (*n* = 37/73)	67.4% (*n* = 29/43)	6 g/day	Lack of demographic and anamnestic data; lack of randomization; info for tolerability only for 20 patients	6 months	118 (73 treated; 45 controls)	Prospective, non-interventional observational study with untreated controls	HR (16, 18, 26, 31, 33, 35, 39, 45, 51, 52, 53, 56, 58, 59, 66, 68, 73, and 82) genotypes
EGCG, FA, B12, and HA [68]	N/A	100% (*n* = 1/1)	2 tabs/day	Need for longer monitoring	8 months	1 treated	Case report	HR (HPV 16) genotype
EGCG, FA, B12, and HA [70]	85% (*n* = 17/20)	85% (*n* = 17/20)	1 tab/day	Sample size; lack of randomization; lack of placebo	3 months	41 (20 treated; 21 controls)	Open-label, controlled (no treatment) clinical trial	HR (HPV 16, 18, 33, 45 and 52) genotypes
EGCG, FA, B12, and HA [69]	100% (*n* = 5/5)	100% (*n* = 5/5)	1 or 2 tab/day	Sample size; lack of randomization and control group	3–6 months	5 (all treated)	Case reports	HR (HPV 16, 18, 31, 51, 45, 52) genotypes
EGCG, FA, B12, and HA [71]	81.4% (*n* = 70/86)	40.7 (*n* = 35/86)	1 tab/day	Lack of randomization; no genotyping or viral load quantification	3 months	163 (86 treated; 77 controls)	Open-label, controlled clinical trial	HR (31, 33, 35, 39, 45, 51, 52, 56, 58, 59, 66, and 68) genotypes
		83.7 (*n* = 72/86)	1 tab/day		6 months			
EGCG, FA, B12, and HA [72]	66.7% (*n* = 14/21)	80% (*n* = 4/5)	1 tab/day	Lack of control; lack of immune biomarker analysis	3 months	106 (all treated)	Single arm, open-label clinical study	HR (16, 18, 31, 33, 35, 39, 45, 51, 52, 56, 58, 59, 66 and 68) genotypes
	85.8% (*n* = 91/106)	92.3% (*n* = 36/39)	1 tab/day		6 months			

For each study the reported outcomes, such as clearance rates and lesion resolution outcomes (expressed in percentage), are summarized, along with the number of patients involved (n), dosage, treatment duration, the reported study limitations, sample size, study design, and HPV genotype. Abbreviation: epigallocatechin gallate (EGCG), folic acid (FA), vitamin B12 (B12), and hyaluronic acid (HA); Low-risk (LR); High-risk (HR); N/A indicates data not available or not reported in the study; ns indicates non-significant relevance.

## 4. Discussion

**Figure 3:**
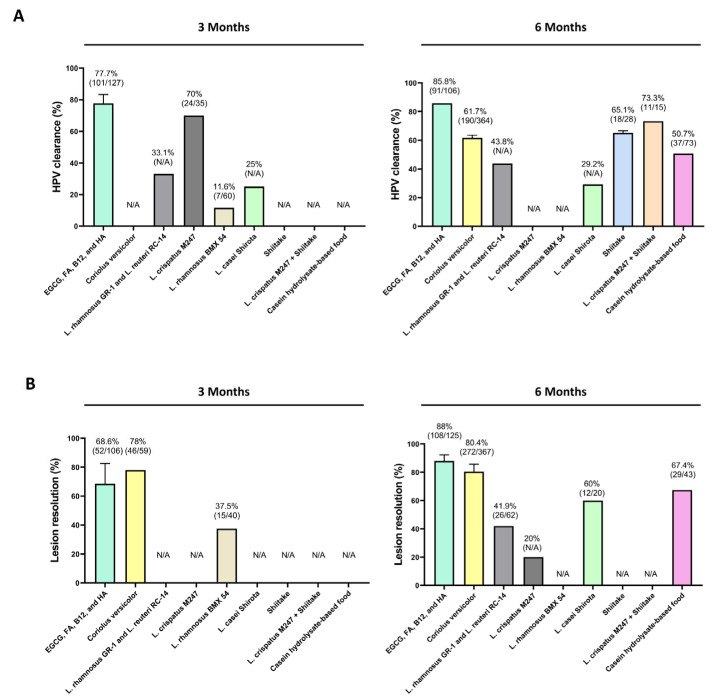
Graphical representation of the effects of dietary supplementation-based and local approaches on HPV clearance (**A**) and cervical lesion resolution (**B**) at the most representative time points, 3- and 6-months post-treatment. For each formulation, data (i.e., total HPV clearance and lesion resolution) from individual studies were averaged, and patient numbers were summed to generate overall estimates for each time point. Detailed values from the individual studies included in this analysis are provided in Table 1. Abbreviation: epigallocatechin gallate (EGCG), folic acid (FA), vitamin B12 (B12), and hyaluronic acid (HA); N/A indicates data not available or not reported in the study.

In this review we provided an overview of the current available dietary supplementation-based and local approaches with clinical relevance for the management of HPV infection, with particular focus on medicinal mushrooms, probiotics, and the combinations of natural molecules (Figure 3).

Among these interventions, the combination of EGCG, FA, B12, and HA exhibited the highest rate of HPV clearance and lesion resolution relative to the number of patients included across studies. Both EGCG, FA, B12, and HA and *Coriolus versicolor* formulations are among the most well-documented interventions based on dietary supplements and local approaches in HPV management. Although both treatments—within the respective clinical studies—demonstrated similar lesion resolution rates (around 92%) after 6 months, the EGCG, FA, B12, HA formulation reported a higher viral clearance rate at the same time point. Notably, such combined natural molecules also achieved statistically significant clearance as early as 3 months, whereas the *Coriolus versicolor*-based approach did not report any measurable effects at that earlier timepoint.

Moreover, the high rate of spontaneous viral clearance is a crucial factor when evaluating HPV treatment outcomes as it may underestimate the effectiveness of the treatment. According to the extrapolated data from previously published studies reported in Porcaro and colleagues’ publication [72], the EGCG, FA, B12, HA formulation exhibited a substantial deviation in HPV clearance from expected spontaneous clearance rates at both 3- and 6-month time points. This suggests that the indicated treatment may accelerate HPV resolution compared to spontaneous regression. In addition, although medicinal mushrooms may act by enhancing the immune surveillance, thus promoting antiviral responses [73], the ability of HPV to escape the immune detection may limit their potential therapeutic effect [74].

Among the other available natural extracts, Praneem polyherbal extract and casein hydrolysate formulations reported interesting results in HPV management. However, each one has been evaluated in only a single study, making it difficult to draw definitive conclusions about their efficacy and safety due to an insufficient amount of scientific evidence.

Finally, the use of probiotics, including *L. crispatus*, *L. gasseri*, and *L. casei*, may contribute to the modulation of the existing communities of vaginal microbiota also enhancing viral clearance through immune modulation, the competitive exclusion of pathogens, and the production of protective metabolites, such as lactic acid. However, evaluated studies lack crucial information regarding the effects of such a probiotic approach in improving HPV infection. For instance, some reports highlighted the effect of probiotics on HPV clearance without assessing concurrent improvement in cervical lesions, or vice versa. Moreover, well-defined time points frequently lack in study design, focusing on follow-up periods that coincide with the window of spontaneous HPV regression. Furthermore, diverse administration regimens, such as multiple daily oral doses or vaginal applications, can impact usability and patient adherence, with more complex or invasive regimens, thus potentially limiting consistent application (see Table 1).

In addition, to strengthen the interpretation of the observed treatment outcomes, several factors should be carefully considered in both current and future research. These include age, type of infection (persistent vs. non-persistent), cytological severity of lesions (LSIL vs. HSIL), HPV genotype (HR vs. LR types), treatment duration, geographic location, lifestyle factors (e.g., smoking and sexual behavior), comorbidities, and host immune status. Stratifying patients by HPV subtype, especially HR genotypes, and analyzing HPV-related biomarkers associated with cancer risk (e.g., anti-HPV16 E6 antibodies, cytokine profiles, RNA sequencing data, microRNA expression) may improve the quality of future research. Besides this, considering randomized clinical trials, long-term safety assessments and including follow-up time points in the study design may corroborate scientific evidence for supporting the use of these interventions into clinical practice, ultimately improving the management of HPV infection.

## 5. Conclusions

HPV infection is a worldwide issue, and its management remains a great challenge. Programs for screening and vaccination are essential for lowering the prevalence and burden of diseases linked to HPV. However, medical practice still lacks a safe and efficient therapeutic approach that targets HPV persistence. In this regard, dietary supplements and local strategies based on probiotics, medicinal mushrooms, and mixed natural molecules showed encouraging outcomes in promoting HPV clearance and enhancing cervical cytological lesions caused by the virus. Every approach that has been considered has advantages and disadvantages that could be related to the intricate pathophysiology of HPV infection.

Methodological limitations in previous research, such as sample size, study design heterogeneity, and variations in dosage and formulation, could account for the variations in observed results between studies. However, the observed variation in clinical outcomes is also driven by the complicated heterogeneous nature of HPV infection itself, which is influenced by individual immunogenetic variability. Among the evaluated strategies, the combination of EGCG, FA, B12, and HA demonstrated promising results in terms of HPV clearance and lesion resolution, with statistically significant effects observed as early as three months. Of course, future research based on larger and randomized clinical studies will contribute to consolidating the reported evidence and provide stronger support for the use of these interventions in the management of HPV infection.

## Abbreviations

The following abbreviations are used in this manuscript:

ASCUS	Atypical Squamous Cells of Undetermined Significance
B12	Vitamin B12
CIN1	Cervical Intraepithelial Neoplasia Grade 1
CIN2/CIN3	Cervical Intraepithelial Neoplasia Grade 2/Grade 3
E6/E7	Oncogenic Proteins E6 and E7
EGCG	Epigallocatechin Gallate
FA	Folic Acid
FDA	Food and Drug Administration
HA	Hyaluronic Acid
HIV	Human Immunodeficiency Virus
HPV	Human Papillomavirus
HR	High-Risk
HSIL	High-Grade Squamous Intraepithelial Lesion
IFN-γ	Interferon-gamma
IL	Interleukin (e.g., IL12)
LEEP	Loop Electrosurgical Excision Procedure
LMICs	Low- and Middle-Income Countries
LR	Low-Risk
LSIL	Low-Grade Squamous Intraepithelial Lesion
N/A	Not applicable
p53	Tumor Suppressor Protein p53
pRb	Retinoblastoma Protein
PSP	Polysaccharopeptides
TNF-α	Tumor necrosis factor alpha

## References

[1] Bartosik M., Moranova L., Izadi N., Strmiskova J., Sebuyoya R., Holcakova J., Hrstka R. (2024). Advanced Technologies towards Improved HPV Diagnostics. J. Med. Virol..

[2] Fashedemi O., Ozoemena O.C., Peteni S., Haruna A.B., Shai L.J., Chen A., Rawson F., Cruickshank M.E., Grant D., Ola O. (2024). Advances in Human Papillomavirus Detection for Cervical Cancer Screening and Diagnosis: Challenges of Conventional Methods and Opportunities for Emergent Tools. Anal. Methods.

[3] Alemany L., Felsher M., Giuliano A.R., Waterboer T., Mirghani H., Mehanna H., Roberts C., Chen Y.T., Lara N., Lynam M. (2025). Oral Human Papillomavirus (HPV) Prevalence and Genotyping among Healthy Adult Populations in the United States and Europe: Results from the PROGRESS (PRevalence of Oral hpv infection, a Global aSSessment) Study. eClinicalMedicine.

[4] Seyoum A., Assefa N., Gure T., Seyoum B., Mulu A., Mihret A. (2022). Prevalence and Genotype Distribution of High-Risk Human Papillomavirus Infection Among Sub-Saharan African Women: A Systematic Review and Meta-Analysis. Front. Public Health.

[5] Krasniqi E., Barba M., Venuti A., Pizzuti L., Cappuzzo F., Landi L., Carpano S., Marchetti P., Villa A., Vizza E. (2021). Circulating HPV DNA in the Management of Oropharyngeal and Cervical Cancers: Current Knowledge and Future Perspectives. J. Clin. Med..

[6] Quinlan J.D. (2021). Human Papillomavirus: Screening, Testing, and Prevention. Am. Fam. Physician.

[7] Yang D., Zhang J., Cui X., Ma J., Wang C., Piao H. (2022). Risk Factors Associated With Human Papillomavirus Infection, Cervical Cancer, and Precancerous Lesions in Large-Scale Population Screening. Front. Microbiol..

[8] Mathur S., Conway D.I., Worlledge-Andrew H., Macpherson L.M.D., Ross A.J. (2015). Assessment and Prevention of Behavioural and Social Risk Factors Associated with Oral Cancer: Protocol for a Systematic Review of Clinical Guidelines and Systematic Reviews to Inform Primary Care Dental Professionals. Syst. Rev..

[9] Keller K., Ramos-Cartagena J.M., Guiot H.M., Muñoz C., Rodríguez Y., Colón-López V., Deshmukh A.A., Tirado-Gómez M., Ortiz A.P. (2022). Association of Smoking with Anal High-Risk HPV Infection and Histologically Confirmed Anal High-Grade Squamous Intraepithelial Lesions among a Clinic-Based Population in Puerto Rico. Cancer Treat. Res. Commun..

[10] Madathil S., Rousseau M.C., Durán D., Alli B.Y., Joseph L., Nicolau B. (2022). Life Course Tobacco Smoking and Risk of HPV-Negative Squamous Cell Carcinomas of Oral Cavity in Two Countries. Front. Oral Health.

[11] Best S.R., Niparko K.J., Pai S.I. (2012). Biology of Human Papillomavirus Infection and Immune Therapy for HPV-Related Head and Neck Cancers. Otolaryngol. Clin. N. Am..

[12] Richardson H., Kelsall G., Tellier P., Voyer H., Abrahamowicz M., Ferenczy A., Coutlée F., Franco E.L. (2003). The Natural History of Type-Specific Human Papillomavirus Infections in Female University Students. Cancer Epidemiol. Biomark. Prev..

[13] Schiffman M., Doorbar J., Wentzensen N., De Sanjosé S., Fakhry C., Monk B.J., Stanley M.A., Franceschi S. (2016). Carcinogenic Human Papillomavirus Infection. Nat. Rev. Dis. Primers.

[14] Brown D.B., Shew M.L., Qadadri B., Neptune N., Vargas M., Tu W., Juliar B.E., Breen T.E., Fortenberry J.D. (2005). A Longitudinal Study of Genital Human Papillomavirus Infection in a Cohort of Closely Followed Adolescent Women. J. Infect. Dis..

[15] Franco E.L., Villa L.L., Sobrinho J.P., Prado J.M., Rousseau M.C., Désy M., Rohan T.E. (1999). Epidemiology of Acquisition and Clearance of Cervical Human Papillomavirus Infection in Women from a High-Risk Area for Cervical Cancer. J. Infect. Dis..

[16] Giuliano A.R., Harris R., Sedjo R.L., Baldwin S., Roe D., Papenfuss M.R., Abrahamsen M., Inserra P., Olvera S., Hatch K. (2002). Incidence, Prevalence, and Clearance of Type-Specific Human Papillomavirus Infections: The Young Women’s Health Study. J. Infect. Dis..

[17] Giuliano A.R., Lee J.H., Fulp W., Villa L.L., Lazcano E., Papenfuss M.R., Abrahamsen M., Salmeron J., Anic G.M., Rollison D.E. (2011). Incidence and Clearance of Genital Human Papillomavirus Infection in Men (HIM): A Cohort Study. Lancet.

[18] Pal A., Kundu R. (2020). Human Papillomavirus E6 and E7: The Cervical Cancer Hallmarks and Targets for Therapy. Front. Microbiol..

[19] Syrjänen S. (2018). Oral Manifestations of Human Papillomavirus Infections. Eur. J. Oral Sci..

[20] Gholamzad A., Khakpour N., Hashemi M., Gholamzad M. (2024). Prevalence of High and Low Risk HPV Genotypes among Vaccinated and Non-Vaccinated People in Tehran. Virol. J..

[21] Mao C., Koutsky L.A., Ault K.A., Wheeler C.M., Brown D.R., Wiley D.J., Alvarez F.B., Bautista O.M., Jansen K.U., Barr E. (2006). Efficacy of Human Papillomavirus-16 Vaccine to Prevent Cervical Intraepithelial Neoplasia: A Randomized Controlled Trial. Obstet. Gynecol..

[22] Cuschieri K.S., Cubie H.A., Whitley M.W., Gilkison G., Arends M.J., Graham C., McGoogan E. (2005). Persistent High Risk HPV Infection Associated with Development of Cervical Neoplasia in a Prospective Population Study. J. Clin. Pathol..

[23] Koshiol J., Lindsay L., Pimenta J.M., Poole C., Jenkins D., Smith J.S. (2008). Persistent Human Papillomavirus Infection and Cervical Neoplasia: A Systematic Review and Meta-Analysis. Am. J. Epidemiol..

[24] Rositch A.F., Koshiol J., Hudgens M.G., Razzaghi H., Backes D.M., Pimenta J.M., Franco E.L., Poole C., Smith J.S. (2013). Patterns of Persistent Genital Human Papillomavirus Infection among Women Worldwide: A Literature Review and Meta-Analysis. Int. J. Cancer.

[25] Shulzhenko N., Lyng H., Sanson G.F., Morgun A. (2014). Ménage à Trois: An Evolutionary Interplay between Human Papillomavirus, a Tumor, and a Woman. Trends Microbiol..

[26] Tainio K., Athanasiou A., Tikkinen K.A.O., Aaltonen R., Cárdenas J., Hernándes, Glazer-Livson S., Jakobsson M., Joronen K., Kiviharju M. (2018). Clinical Course of Untreated Cervical Intraepithelial Neoplasia Grade 2 under Active Surveillance: Systematic Review and Meta-Analysis. BMJ.

[27] Huber J., Mueller A., Sailer M., Regidor P.A. (2021). Human Papillomavirus Persistence or Clearance after Infection in Reproductive Age. What Is the Status? Review of the Literature and New Data of a Vaginal Gel Containing Silicate Dioxide, Citric Acid, and Selenite. Women’s Health.

[28] Palefsky J. (2006). CHAPTER 5 HPV Infection and HPV-Associated Neoplasia in Immunocompromised Women. Int. J. Gynecol. Obstet..

[29] Kilic D., Guler T., Atigan A., Avsaroglu E., Karakaya Y.A., Kaleli I., Kaleli B. (2020). Predictors of Human Papillomavirus (HPV) Persistence after Treatment of High Grade Cervical Lesions; Does Cervical Cytology Have Any Prognostic Value in Primary HPV Screening?. Ann. Diagn. Pathol..

[30] Abate A., Munshea A., Nibret E., Alemayehu D.H., Alemu A., Abdissa A., Mihret A., Abebe M., Mulu A. (2025). Persistence and Clearance Rates of Human Papillomaviruses in a Cohort of Women Treated or Not Treated for Cervical Dysplasia in Northwest Ethiopia. Sci. Rep..

[31] Laganà A.S., Chiantera V., Gerli S., Proietti S., Lepore E., Unfer V., Carugno J., Favilli A. (2023). Preventing Persistence of HPV Infection with Natural Molecules. Pathogens.

[32] Gardella B., Gritti A., Soleymaninejadian E., Pasquali M.F., Riemma G., La Verde M., Schettino M.T., Fortunato N., Torella M., Dominoni M. (2022). New Perspectives in Therapeutic Vaccines for HPV: A Critical Review. Medicina.

[33] Yousefi Z., Aria H., Ghaedrahmati F., Bakhtiari T., Azizi M., Bastan R., Hosseini R., Eskandari N. (2022). An Update on Human Papilloma Virus Vaccines: History, Types, Protection, and Efficacy. Front. Immunol..

[34] Saleh M.H., Rashedi I., Keating A. (2017). Immunomodulatory Properties of Coriolus Versicolor: The Role of Polysaccharopeptide. Front. Immunol..

[35] Donatini B. (2014). Control of Oral Human Papillomavirus (HPV) by Medicinal Mushrooms, Trametes Versicolor and Ganoderma Lucidum: A Preliminary Clinical Trial. Int. J. Med. Mushrooms.

[36] Criscuolo A.A., Sesti F., Piccione E., Mancino P., Belloni E., Gullo C., Ciotti M. (2021). Therapeutic Efficacy of a Coriolus Versicolor-Based Vaginal Gel in Women with Cervical Uterine High-Risk HPV Infection: A Retrospective Observational Study. Adv. Ther..

[37] Serrano L., López A.C., González S.P., Palacios S., Dexeus D., Centeno-Mediavilla C., Coronado P., De La Fuente J., López J.A., Vanrell C. (2021). Efficacy of a Coriolus Versicolor-Based Vaginal Gel in Women with Human Papillomavirus-Dependent Cervical Lesions: The PALOMA Study. J. Low. Genit. Tract. Dis..

[38] Gil-Antuñano S.P., Serrano Cogollor L., López Díaz A.C., González Rodríguez S.P., Dexeus Carter D., Centeno Mediavilla C., Coronado Martín P., de la Fuente Valero J., López Fernández J.A., Vanrell Barbat C. (2022). Efficacy of a Coriolus Versicolor-Based Vaginal Gel in Human Papillomavirus-Positive Women Older Than 40 Years: A Sub-Analysis of PALOMA Study. J. Pers. Med..

[39] Cortés Bordoy J., de Santiago García J., Agenjo González M., Dexeus Carter D., Fiol Ruiz G., García Ferreiro C., González Rodríguez S.P., Gurrea Soteras M., Martínez Lamela E., Palacios Gil-Antuñano S. (2023). Effect of a Multi-Ingredient Coriolus-Versicolor-Based Vaginal Gel in Women with HPV–Dependent Cervical Lesions: The Papilobs Real-Life Prospective Study. Cancers.

[40] Kajiyama S., Nagatake T., Ishikawa S., Hosomi K., Shimada Y., Matsui Y., Kunisawa J. (2023). Lentinula Edodes Mycelia Extract Regulates the Function of Antigen-Presenting Cells to Activate Immune Cells and Prevent Tumor-Induced Deterioration of Immune Function. BMC Complement. Med. Ther..

[41] Kuroki T., Lee S., Hirohama M., Taku T., Kumakura M., Haruyama T., Nagata K., Kawaguchi A. (2018). Inhibition of Influenza Virus Infection by Lentinus Edodes Mycelia Extract through Its Direct Action and Immunopotentiating Activity. Front. Microbiol..

[42] Bugajewski M., Angerhoefer N., Pączek L., Kaleta B. (2025). Lentinula Edodes as a Source of Bioactive Compounds with Therapeutical Potential in Intestinal Inflammation and Colorectal Cancer. Int. J. Mol. Sci..

[43] Smith J.A., Mathew L., Gaikwad A., Rech B., Burney M.N., Faro J.P., Lucci J.A., Bai Y., Olsen R.J., Byrd T.T. (2019). From Bench to Bedside: Evaluation of AHCC Supplementation to Modulate the Host Immunity to Clear High-Risk Human Papillomavirus Infections. Front. Oncol..

[44] Smith J.A., Gaikwad A.A., Mathew L., Rech B., Faro J.P., Lucci J.A., Bai Y., Olsen R.J., Byrd T.T. (2022). AHCC^®^ Supplementation to Support Immune Function to Clear Persistent Human Papillomavirus Infections. Front. Oncol..

[45] Alizhan D., Ukybassova T., Bapayeva G., Aimagambetova G., Kongrtay K., Kamzayeva N., Terzic M. (2025). Cervicovaginal Microbiome: Physiology, Age-Related Changes, and Protective Role Against Human Papillomavirus Infection. J. Clin. Med..

[46] Lin F.-C., Young H.A. (2014). Interferons: Success in Anti-Viral Immunotherapy. Cytokine Growth Factor. Rev..

[47] Zeng M., Li X., Jiao X., Cai X., Yao F., Xu S., Huang X., Zhang Q., Chen J. (2023). Roles of Vaginal Flora in Human Papillomavirus Infection, Virus Persistence and Clearance. Front. Cell Infect. Microbiol..

[48] Brotman R.M., Shardell M.D., Gajer P., Tracy J.K., Zenilman J.M., Ravel J., Gravitt P.E. (2014). Interplay between the Temporal Dynamics of the Vaginal Microbiota and Human Papillomavirus Detection. J. Infect. Dis..

[49] Brusselaers N., Shrestha S., van de Wijgert J., Verstraelen H. (2019). Vaginal Dysbiosis and the Risk of Human Papillomavirus and Cervical Cancer: Systematic Review and Meta-Analysis. Am. J. Obstet. Gynecol..

[50] Papamentzelopoulou M., Pitiriga V.C. (2025). Unlocking the Interactions Between the Whole-Body Microbiome and HPV Infection: A Literature Review. Pathogens.

[51] Reid G., Abrahamsson T., Bailey M., Bindels L.B., Bubnov R., Ganguli K., Martoni C., O’Neill C., Savignac H.M., Stanton C. (2017). How Do Probiotics and Prebiotics Function at Distant Sites?. Benef. Microbes.

[52] Wang Z., Wang Q., Zhao J., Gong L., Zhang Y., Wang X., Yuan Z. (2019). Altered Diversity and Composition of the Gut Microbiome in Patients with Cervical Cancer. AMB Express.

[53] Di Pierro F., Criscuolo A.A., Dei Giudici A., Senatori R., Sesti F., Ciotti M., Piccione E. (2021). Oral Administration of Lactobacillus Crispatus M247 to Papillomavirus-Infected Women: Results of a Preliminary, Uncontrolled, Open Trial. Minerva Obstet. Gynecol..

[54] Dellino M., Cascardi E., Laganà A.S., Di Vagno G., Malvasi A., Zaccaro R., Maggipinto K., Cazzato G., Scacco S., Tinelli R. (2022). Lactobacillus Crispatus M247 Oral Administration: Is It Really an Effective Strategy in the Management of Papillomavirus-Infected Women?. Infect. Agent. Cancer.

[55] Verhoeven V., Renard N., Makar A., Van Royen P., Bogers J.P., Lardon F., Peeters M., Baay M. (2013). Probiotics Enhance the Clearance of Human Papillomavirus-Related Cervical Lesions: A Prospective Controlled Pilot Study. Eur. J. Cancer Prev..

[56] Segers M.E., Lebeer S. (2014). Towards a Better Understanding of Lactobacillus Rhamnosus GG—Host Interactions. Microb. Cell Fact..

[57] Palma E., Recine N., Domenici L., Giorgini M., Pierangeli A., Panici P.B. (2018). Long-Term Lactobacillus Rhamnosus BMX 54 Application to Restore a Balanced Vaginal Ecosystem: A Promising Solution against HPV-Infection. BMC Infect. Dis..

[58] Mu Q., Tavella V.J., Luo X.M. (2018). Role of Lactobacillus Reuteri in Human Health and Diseases. Front. Microbiol..

[59] Ou Y.C., Fu H.C., Tseng C.W., Wu C.H., Tsai C.C., Lin H. (2019). The Influence of Probiotics on Genital High-Risk Human Papilloma Virus Clearance and Quality of Cervical Smear: A Randomized Placebo-Controlled Trial. BMC Womens Health.

[60] Salimbeni V., Martinelli C., Porto L., Le Donne M., Alibrandi A., Di Pierro F., Cau F., Granese R., Iannone V., Marchetta L. (2024). A Prospective Observational Study to Evaluate Impact of Oral Supplementation with AHCC and Lactobacillus Crispatus M247 on HPV Clearance and Low-Grade Squamous Intraepithelial Lesion Regression. Ann. Res. Oncol..

[61] Mitra A., Gultekin M., Burney Ellis L., Bizzarri N., Bowden S., Taumberger N., Bracic T., Vieira-Baptista P., Sehouli J., Kyrgiou M. (2024). Genital Tract Microbiota Composition Profiles and Use of Prebiotics and Probiotics in Gynaecological Cancer Prevention: Review of the Current Evidence, the European Society of Gynaecological Oncology Prevention Committee Statement. Lancet Microbe.

[62] Shukla S., Bharti A.C., Hussain S., Mahata S., Hedau S., Kailash U., Kashyap V., Bhambhani S., Roy M., Batra S. (2009). Elimination of High-Risk Human Papillomavirus Type HPV16 Infection by “Praneem” Polyherbal Tablet in Women with Early Cervical Intraepithelial Lesions. J. Cancer Res. Clin. Oncol..

[63] Pingarron C., Duque A., López A., Ferragud J. (2025). A Prospective, Non-Interventional Observational Study to Assess the Efficacy, Safety, and Tolerability of the Casein Hydrolysate-Based Food Supplement in High-Risk Human Papillomavirus-Positive Women. Cureus.

[64] Chen L.M., Cong Q., Wu D., Chen Y., Qiu L.H., Hong Z.B., Yang Y.B., Xu L., Wang L.F., Huang L.X. (2023). A Prospective Multicentre Controlled Study of Gaoweikang (Chinese Multiherb Extract-Based Tincture) Used in High-Risk HPV Infections. Eur. Rev. Med. Pharmacol. Sci..

[65] Kiyoshima C., Kimura I., Ishida K., Hirano T., Ishida T., Shigekawa K., Yoshikawa K., Yotsumoto F. (2025). Effectiveness of the Traditional Japanese Herbal Medicine, Yokuinin (Kampo), in the Treatment of Cervical Precancerous Lesions. Cureus.

[66] Wang Y.Q., Lu J.L., Liang Y.R., Li Q.S. (2018). Suppressive Effects of EGCG on Cervical Cancer. Molecules.

[67] Frega A., Gentili C., Proietti S., Lepore E., Unfer V., Fuso A. (2023). Epigallocatechin Gallate, Folic Acid, Vitamin B12, and Hyaluronic Acid Significantly Increase Apoptosis and P53 Expression in HeLa Cells. Eur. Rev. Med. Pharmacol. Sci..

[68] Grandi G., Botticelli L., Fraia P.D., Babalini C., Masini M., Unfer V. (2023). The Association of Four Natural Molecules—EGCG, Folic Acid, Vitamin B12, and HA—To Counteract HPV Cervical Lesions: A Case Report. J. Pers. Med..

[69] Calcagno M., Incocciati B., Di Fraia L., Unfer V. (2024). Counteracting HPV Cervical and Anal Infection through Dietary Supplementation of EGCG, Folic Acid, Vitamin B12 and Hyaluronic Acid: Clinical Case Reports. J. Clin. Med..

[70] Aragona C., Bezerra Espinola M.S., Bilotta G., Porcaro G., Calcagno M. (2023). Evaluating the Efficacy of Pervistop^®^, a New Combination Based on EGCG, Folic Acid, Vitamin B12 and Hyaluronic Acid on Patients with Human Papilloma Virus (HPV) Persistent Infections and Cervical Lesions: A Pilot Study. J. Clin. Med..

[71] Tinelli A., Gustapane S., Licchelli M., Coluccia A.C., Panese G., Proietti S., Gambioli R. (2024). Treatment with Epigallocatechin Gallate, Folic Acid, Vitamin B12, and Hyaluronic Acid Decreases HPV Positivity in Women Attending Regional Screening in Puglia. Microorganisms.

[72] Porcaro G., Pavone-Cossut M.R., Moretti S., Bilotta G., Aragona C., Unfer V. (2025). Oral Treatment with EGCG, Folic Acid, Vitamin B12, and Hyaluronic Acid Improves HPV Clearance and Counteracts Its Persistence: A Clinical Study. Int. J. Mol. Sci..

[73] Rokos T., Pribulova T., Kozubik E., Biringer K., Holubekova V., Kudela E. (2023). Exploring the Bioactive Mycocompounds (Fungal Compounds) of Selected Medicinal Mushrooms and Their Potentials against HPV Infection and Associated Cancer in Humans. Life.

[74] Senba M., Mori N. (2012). Mechanisms of Virus Immune Evasion Lead to Development from Chronic Inflammation to Cancer Formation Associated with Human Papillomavirus Infection. Oncol. Rev..

